# Ontology design patterns to disambiguate relations between genes and gene products in GENIA

**DOI:** 10.1186/2041-1480-2-S5-S1

**Published:** 2011-10-06

**Authors:** Robert Hoehndorf, Axel-Cyrille Ngonga Ngomo, Sampo Pyysalo, Tomoko Ohta, Anika Oellrich, Dietrich Rebholz-Schuhmann

**Affiliations:** 1 European Bioinformatics Institute, Hinxton, Cambridge, UK; 2 Department of Computer Science, University of Leipzig, Leipzig, Germany; 3 Department of Computer Science, University of Tokyo, Tokyo, Japan; 4 Department of Genetics, University of Cambridge, UK

## Abstract

**Motivation:**

Annotated reference corpora play an important role in biomedical information extraction. A semantic annotation of the natural language texts in these reference corpora using formal ontologies is challenging due to the inherent ambiguity of natural language. The provision of formal definitions and axioms for semantic annotations offers the means for ensuring consistency as well as enables the development of verifiable annotation guidelines. Consistent semantic annotations facilitate the automatic discovery of new information through deductive inferences.

**Results:**

We provide a formal characterization of the relations used in the recent GENIA corpus annotations. For this purpose, we both select existing axiom systems based on the desired properties of the relations within the domain and develop new axioms for several relations. To apply this ontology of relations to the semantic annotation of text corpora, we implement two ontology design patterns. In addition, we provide a software application to convert annotated GENIA abstracts into OWL ontologies by combining both the ontology of relations and the design patterns. As a result, the GENIA abstracts become available as OWL ontologies and are amenable for automated verification, deductive inferences and other knowledge-based applications.

**Availability:**

Documentation, implementation and examples are available from http://www-tsujii.is.s.u-tokyo.ac.jp/GENIA/.

## Background

The goal of Information Extraction (IE) is to recognize specific pieces of information in natural language texts and to represent them in a structured form that comprises meaningful associations of relevant entities. For this reason, IE approaches typically involve Named Entity Recognition (NER) where *mentions* of specific types of “real-world” entities, such as people or places, are detected in text. To facilitate reliable biomedical IE, considerable efforts have been made with regard to the development of specialized NER methods for key domain entities, focusing in particular on the recognition of gene and gene product (GGP) mentions [1-3]. As GGP mentions can further be normalized to identify specific entries in databases such as UniProt, they provide a connection to entities relevant to biomolecular research and thus a solid basis for domain IE. However, in contrast to the well-defined meaning of the basic entities, the semantics of their associations are often only informally defined.

In biomedical IE, extracted information is frequently represented simply as untyped pairs of entities representing, for instance, protein-protein or gene-disease associations [4]. However, even resources identifying protein-protein interactions as entity pairs diverge considerably in their actual annotations [5], leading to restrictions ranging from usability to interpretability of both the annotations and IE results. In response to the limitations of such representations, there has recently been increased interest in richer representations of extracted information [6] and a number of corpora have been published that annotate associations between entities by using fine-grained types drawn from ontologies [7,8]. Yet, no definition or axiomatization of these relations has been proposed so far. Definitions and axioms are necessary to make the *meaning* of the relations explicit, and to provide the means for developing consistent and verifiable annotation guidelines allowing for the automatic detection of inconsistent annotations and enabling the discovery of new information through deductive inferences. Here, our aim is to define such relations and axioms for fundamental relations such as *part-of* connecting GGPs to referents of non-specific domain terms such as *promoter region*. Annotations to these fundamental relations have been introduced recently [9,10] to the widely used GENIA corpus [11].

Providing formal definitions and axioms for these relations is challenging because the relation annotations are based on the use of the relations in text, where it is generally not possible to enforce a common understanding of terms. We extend our preliminary work [12] and present a formal characterization of the relations used in the GENIA relationship annotation based on two ontology design patterns. These patterns are not restricted to an application within the GENIA corpus annotation, but can be applied in a wide number of domains, in particular in ontology- and knowledge-based applications using the categories of biological sequences, DNA, RNA or proteins. We implement the developed formalisms in OWL and provide a conversion software to represent annotated GENIA abstracts in OWL.

### The GENIA corpus

The GENIA corpus consists of 2,000 PubMed abstracts annotated manually by biomedical domain experts as a resource for the development and evaluation of domain information extraction (IE) methods. GENIA is one of the most widely used corpora for biomedical IE and has served as the basis for two community-wide shared tasks on named entity recognition [1] and event extraction [6]. The annotations of the corpus abstracts include markup that identifies occurrences of domain terms and named entities, as well as statements of events involving these terms and entities [8,11,13]. The most recent addition to the corpus annotations covers relations between references to named entities and other domain terms [9].

### Formal ontology

An ontology is the formal specification of a conceptualization of a domain [14]. A conceptualization is a system of classes accounting for a particular view on the world [15]. Ontologies are used to specify the meaning of terms within a vocabulary. A basic ontological distinction is made between classes and individuals (or particulars). A *class* is an entity that can be predicated of other entities and that can have instances. The **instance-of** relation links *instances* to the *class* of which they are an instance. Some instances may be classes themselves and have further instances while an *individual* is an entity that cannot be further instantiated [16].

For the purpose of formalizing the relations used in the GENIA corpus, we make use of several biomedical domain ontologies: the Information Artifact Ontology (http://code.google.com/p/information-artifact-ontology/) (IAO), the Sequence Ontology (SO) [17], the Ontology of Biomedical Investigations (OBI) [18], the Gene Ontology (GO) [19] and the GENIA term ontology [11].

Relations in biomedical ontologies can be asserted both between classes and between individuals [20]. Relations between individuals are used to define the relations between classes. These definitions may take the form of reusable patterns, and we will create such patterns for relations between classes in GENIA.

### Preliminaries of GENIA corpus annotation

The first question we have to answer before we can formalize relations used in corpus annotation is what kind of entities are connected through relations in GENIA. Our first observation is that relations in corpus annotations are usually asserted between names and other biomedical domain terms, i.e., between strings that are identified as referring to some kind of entity. While the description of experiments in scientific publications will commonly refer to collections of individuals and not to classes [21], the goal of *named entity recognition* is, among others, the identification of the class to which the characterized collections belong. Therefore, we assume that the names identified in the GENIA corpus denote classes.

In some cases, there is ambiguity in determining the referent of a name or domain term, i.e., certain terms may not refer to identical entities, yet their referents are regarded as *indistinguishable* within the context of a task such as the annotation or recognition of named entities. Regarding certain referents as indistinguishable can improve the automatic extraction of relations and entities. The indistinguishability assumption also allows the definition of generic relations that hold between disjoint classes. Through these means, the effort to create annotation can be reduced, while the applicability of the relations in different tasks and the feasibility of automatic extraction can be maximized. Within GENIA annotations [13] and the NER systems based on it, genes and gene products are not distinguished.

Therefore, a basic precursor for our work is an equivalence relation which states that, within the context of a named entity annotation task, two classes are considered to be *indistinguishable*.

## Results

### Equivalence

Names or terms referring to either a class of genes, DNA, proteins, RNAs and their splice variants, gene products, arbitrary transcripts or similar are considered to be equivalent within the context of the GENIA relation annotations. These classes are called *genes/gene products* (GGPs). For example, *CD19*, *CD19 protein* and *CD19 gene* may be considered to be *equivalent* and represent a single GGP.

We define a class *G_C_* based on a class *C*, which is assumed to be a subclass of *DNA*, and entities derived from *C* through chains of transcription and translation relations *between individuals*. The classes *Protein*, *DNA* and *RNA* are those used in the GENIA term ontology.(1)

Such a formalization has the benefit of connecting the different kinds of GGPs through formal relations that can be exploited by an automated reasoner.

For example, the name “CD19 protein” refers to a class of proteins, and instances of this class stand in a *translated-from* relation to instances of a class of RNA which may be referred to as “CD19 RNA”. Instances of this class of RNA stand in a *transcribed-from* relation to instances of a class of DNA which may be referred to as “CD19 gene”. Thus, according to our definition, all three classes are subclasses of the GGP class *G_CD_*_19_.

### Subclass

The *class-subclass* relation is used to annotate the relation between terms or names in the GENIA corpus where one term refers to a more general class than the other term. For example, this relation holds between the names “CD19 human” (denoting the class *CD19 human*) and “CD19” (denoting a class that is indistinguishable from the class *CD19* (*GGP*)). We base the definition of the *class-subclass* relation upon the ontological *is-a* relation [22]: the classes *C* and *D* stand in the *is-a* relation, if and only if, every instance of *C* is also an instance of *D*.

For example, the referent of the name “human CD19 gene” (the class *CD19 human gene*) stands in the *is-a* relation to the referent of the name “CD19” (the GGP class *CD19* (*GGP*)), because all instances of *CD19 human gene* are also instances of *CD19* (*GGP*).

### Mereological relations

The largest group of relations in the relationship annotations of the GENIA corpus refers to mereological relations, i.e., relations between parts and their wholes. Three kinds of parthood relations are distinguished within GENIA:

• relations between a whole and its components, for example between the classes *CD19 promoter* and *CD19*,

• relations between a collection and its members, as between *Hox gene family* and *HOXA1*,

• the relation between an entity and the location at which this entity exists, such as *CD19* which is located at *CD19 locus.*

Substantial work has already been undertaken with regard to mereological relations and their representation in OWL and biomedical ontologies [20,23,24]. In particular, the relation *CC-part-of*, as a relation between classes (we generally prefix relations between two classes with *CC-*, and relations that hold between two individuals with *II-.*), must be defined in terms of another relation *II-part-of* which is a relation between individuals [20,25]. For example, **CC-part-of** can be defined as *C* ⊑ ∃*II-part-of.D* and **CC-has-part** as *C* ⊑ ∃*II-has-part.D.* Although these definitions are valid for many of the parthood relations asserted between classes in biological ontologies, they are inadequate schemata for parthood relations which have a GGP class as argument, because the GGP class is “too general”.

However, as a GGP class has several *GGP-equivalent* subclasses, the *CC-has-part* and *CC-part-of* relations may be valid for one of these classes but not for the others. For example, assuming the definition of *CC-has-part* above, asserting a *CC-has-part* relation between the GGP class *CD19* (*GGP*) and *CD19 promoter* would be incorrect, because the GGP class will also include the *CD19 protein* class, which has no promoter as part (in virtue of being a class of proteins). Similarly, although it would be correct to assert that *CD19 promoter CC-part-of CD19*, it would be incorrect to say that *CD19 CC-part-of CD19/CD21/CD81/Leu-13 complex.* If the two statements above would hold, we could infer that *CD19 promoter* is *CC-part-of* the *CD19/CD21/CD81/Leu-13 complex*, which is incorrect because protein complexes have no promoters as part.

Consequently, we use the following alternative definition for the *GGP-subclass-has-part* relation (where the argument *G_C_* refers to a GGP class, and *X* to an arbitrary class):(2)

In the OWL syntax, a disjunction of axioms is not permitted. Consequently, we have to reformulate the right side of the definition by using a single subclass axiom (where ⊥ refers to the OWL class owl:Nothing) and derive the equivalent definition:(3)

Intuitively, this definition states that if the GGP class *G_C_* stands in the *GGP-subclass-has-part* relation to the class *X*, then either the DNA, RNA or Protein subclass of *G_C_* must stand in a *CC-has-part* relation to *X*. Using this pattern, we are further able to define the relation *GGP-subclass-part-of* by replacing *II-has-part* with *II-part-of* in definition 3.

*II-part-of* is a primitive relation and we assert axioms that hold for it. *II-part-of* is reflexive, transitive and antisymmetric. We define *II-proper-part-of*:(4)

It is the *II-proper-part-of* relation which will provide the basis for the mereological relations within the GENIA, because identical (or co-extensional) classes are not annotated as standing in a parthood relation. Parthood relations that are not based upon location are further distinguished into two kinds in the GENIA relation annotation: a relation between components and the objects of which they are components, and membership in collections. We assume that the component-object relation (between individuals) *II-oc-part-of* is similar to the relation of determinate parthood [23] in that it is reflexive, transitive, antisymmetric and satisfies the strong supplementation principle [24]. Assuming these axioms for *II-oc-part-of* provides compatibility with the SO, which also assumes the axioms of extensional mereology for the entities classified by it [17,26].

The member-component relation, on the other hand, is a relation between entities of different kinds and is neither reflexive nor antisymmetric [23,27]. The *II-member-of* relation is a sub-relation of the *II-proper-part-of* relation and is non-reflexive, asymmetric and non-transitive [27]. *II-member-of* is not the same relation as the *member-of* relation in the SO; in the SO, *member-of* is transitive, while *II-member-of* is non-transitive. The relation *GGP-subclass-member-of* holds between a GGP class and a collection, such that for one of the subclasses of the GGP class, all instances are a member of some instance of the collection. Therefore, the same pattern as in definition 3 applies for the definition of *GGP-member-of*. For example, the *Lck* (*GGP*) class stands in the *GGP-member-of* relation to the protein family *Src family*, because there is a subclass of *Lck* (*GGP*), i.e., *Lck protein*, such that all instances of this subclass stand in an *II-member-of* relation to some instances of *Src family*. We do not provide a formal characterization of *protein family* here, but re-use the class from the GENIA term ontology and represent specific protein families (such as the *Src family*) as subclasses of GENIA’s *Protein family* class. A detailed formal characterization of *Protein family* within GENIA is subject to future work.

The third parthood relation used in the GENIA corpus annotations is *GGP-subclass-region-of*, which we define by using the primitive *II-region-of* relation. In the GENIA relation annotations, *GGP-subclass-region-of* is used to relate a GGP class to a genomic location. We introduce *GGP-subclass-region-of* to relate the GGP class to the class of loci. The region is a place where all instances of one subclass of the GGP class are located. As for the definition of *GGP-subclass-has-part*, *GGP-subclass-part-of* and *GGP-subclass-member-of*, we assume that there is a subclass of the GGP class for which all instances are located in some instance of the locus, and we use the same pattern as in formula 3. Next we define the interactions of *II-region-of* with *II-part-of*. We want to be able to infer that if the individual *x* is part of *y*, and *y* is located at *z*, then *x* is located at *z*. Furthermore, if the individual *x* is located at *y* and *y* is a part of *z*, then we infer that *x* is located at *z*. We state these conditions using the following axioms in OWL:(5)(6)

### Objects and their variants

The second major group of GENIA corpus relations connects names of GGP classes to names of classes of their variants. Again, we formalize the relations that hold between the classes that are *denoted* by these names.

The GENIA annotations for GGP classes and their variants use six different relations to express the following relationships:

• GGPs to modified proteins, e.g., *TR alpha 1* (*GGP*) to *35S-TR alpha 1* (*Protein*),

• GGPs to protein isoforms, e.g., *ACTA1* (*Protein*) to *G-Actin* (*GGP*),

• GGPs to mutants, e.g., *TNFRI* (*GGP*) to *dominant-negative mutant TNFRI* (*Protein*),

• GGPs to recombinants, e.g., *Oct-2* (*GGP*) to *Oct-2 expression vector* (*DNA*),

• GGPs to precursors, e.g., *IL-16* (*GGP*) to *pro-IL-16* (*Protein*),

• GGPs to experimental material, in particular to antisense elements, e.g., *GATA-3* (*GGP*) to *antisense GATA-3 RNA* (*RNA*).

We call the basic relation between a GGP and its variant *GGP-has-variant*. There is a general schema involved in the sub-relations of *GGP-has-variant* that we exploit in its definition: whenever *GGP-has-variant*(*G_C_*, *D*), then every instance of *D* is a variation of some instance of *G_C_*. Although it is possible to identify a more specific subclass of *G_C_* in some cases, this is not true for all sub-relations of *GGP-has-variant*. We define the relation *G_C_ GGP-has-variant D* by using the relation *II-has-variant*, which is a relation between individuals:(7)

Again, we provide basic axioms for the *II-has-variant* relation. Our first observation is that variance is reflexive, i.e., everything (every molecule) is a variant of itself. Furthermore, variance is symmetric, i.e., if *x* is a variant of *y*, then *y* is a variant of *x*. Whether *II-has-variant* is transitive is more difficult to ascertain. While it seems to be the case that, if *x* is a variant of *y* and *y* a variant of *z*, then *x* is a variant of *z*, this principle may fail if the distance between *x* and *z* increases, i.e., more intermediate variants are introduced. Consequently, we do not assume that *II-has-variant* is transitive.

To formalize a sub-relation of *II-has-variant*, e.g., *II-has-isoform*, we note domain and range of the relation as well as basic axioms. In the definition of the GGP relation, we must carefully consider whether the relation holds between all instances of the GGP class, or only one of its subclasses. For example, the definition of *GGP-has-isoform* between *G_C_* and *D* is:(8)

The relations **GGP-has-recombinant, GGP-has-precursor** and **GGP-has-modified-protein** follow the same pattern.

*II-has-mutant* is a relation between an instance of a GGP class and a mutant of this instance. The relation *II-has-mutant* is irreflexive and symmetric, and consequently not transitive. The definition of *G_C_ GGP-has-mutant D* is as follows:(9)

*II-has-experimental-material* relates an instance of a GGP class to experimental material such as an antisense element. The formal characterization is subject to future work and requires integration with ontologies of experiments such as the Ontology of Biomedical Investigations (OBI) [18].

## Implementation

**Figure 1:**
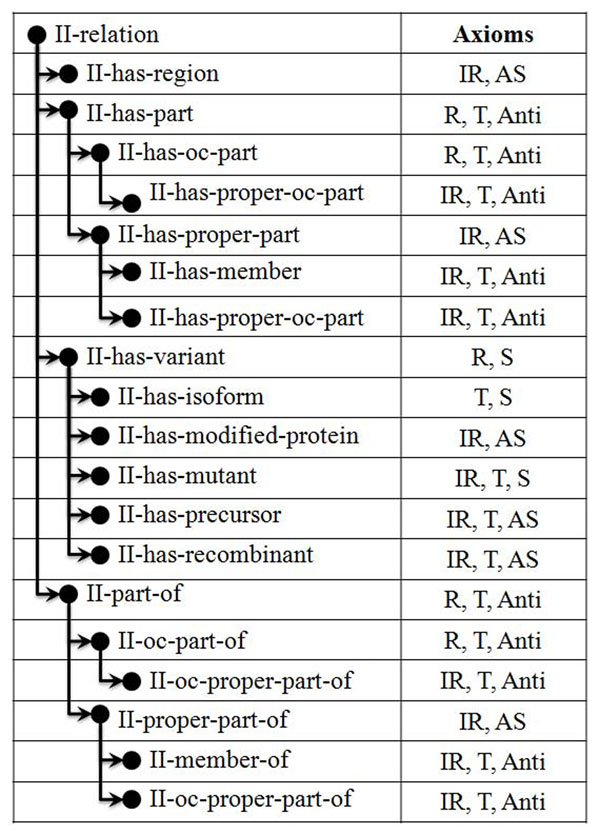
Axioms for the relations in the GENIA relation ontology. R stands for reflexivity, IR for irreflexivity, S for symmetry, T for transitivity, Anti for antisymmetry and AS for asymmetry.

To support automatic inferences and verifications, we provide an implementation which consists of two parts. The first part covers the integration of the basic axioms of relations between individuals into an OWL ontology. It formalizes GENIA’s relation ontology and provides the taxonomy of relations as illustrated in figure 1. To be applicable for automated inferences, we had to omit axioms pertaining to reflexivity or symmetry from the OWL ontology, as those are not permitted for non-primitive properties [28]. The OWL ontology contains the hierarchy of relations and a single new OWL class, the class *GGP*. Furthermore, to provide the definitions of the relations, we also import the OWL versions of the Sequence Ontology (SO) [17] and the GENIA term ontology [29] so that we can refer to relations such as *transcribes-into* from the SO, and to classes such as *DNA* or *Protein* from the GENIA term ontology. The second part provides a conversion from the relations between names and terms that refer to classes in OWL. It is a prototypical conversion tool that translates annotated GENIA abstracts into an OWL file based on the definitions we provide for GENIA’s relationship annotations. The resulting OWL file is based on GENIA’s relation ontology. The conversion tool implements the ontology design patterns we have developed to define relations that take a GGP class as an argument. The conversion tool and examples of converted abstracts are available on the project website at http://www-tsujii.is.s.u-tokyo.ac.jp/GENIA/home/wiki.cgi?page=Relation+annotation.

## Discussion

### Related work

The BioTop Ontology [30] is derived from the GENIA term ontology and provides definitions and axioms for the classes in the GENIA ontology. Additionally, this ontology includes several relations. Some of these relations overlap with those used in the GENIA relation annotation and in the relation ontology, in particular the mereological relations. Yet, BioTop includes mostly the generic definitions of mereological relations. Thus, BioTop’s formalization of mereological relations cannot be used with respect to GGP, as their axioms do not always hold for GGPs as shown earlier. Furthermore, the BioTop ontology does not include any of the variance relations. As BioTop provides a rich axiom system for the classes of the GENIA term ontology, we aim at integrating the BioTop ontology with the relation ontology and the design patterns we provide in future work.

Another relevant ontology is the Gene Regulation Ontology (GRO) [31], which is an ontology for the domain of gene regulation. It provides axioms and definitions for the classes DNA, RNA and protein. Furthermore, it establishes relations between these classes. Therefore, it provides a means for a more detailed specification of GGP classes. GRO does not cover the relations formalized in this work. Rather, it could be allow to provide a more fine-grained definition of GGP classes if necessary.

### Applications in GENIA

There are several applications of formalized relations within the GENIA corpus:

• development of unambiguous annotator guidelines,

• verification of annotations,

• inference of hidden knowledge and

• abductive reasoning, inductive logic programming, rule learning.

Firstly, the development of clear annotator guidelines can be facilitated to increase inter-annotator consistency through the provision of less ambiguity. For this purpose, high expressivity is necessary to specify the meanings of relationship terms or other terms as precisely as possible. To proceed towards the goal of unambiguous, formal guidelines for corpus annotation, we used predicate logic for the formalization, and additionally associated our definitions and axioms with explanations in natural language.

Secondly, the axioms provide a means to *verify* annotations. Such a verification is made possible because axioms restrict the combinations of relations and may lead to contradictions which are sometimes automatically detectable. In particular, the OWL implementation of both the axioms and the ontology design patterns is amenable to automated reasoning and can be used to detect inconsistencies.

Additionally, it is possible to draw inferences from the asserted knowledge automatically. These inferences can be used to verify whether or not erroneous annotations have been asserted by identifying undesired or false inferences. Moreover, automatic inferences can be used to infer hidden or new knowledge.

The conversion tool we provide converts annotated GENIA abstracts into an OWL ontology. This conversion is a form of ontology induction or ontology generation. The resulting ontologies – each covering a domain described within one abstract – can be used for abductive or inductive logic programming, rule learning or other knowledge-based machine learning techniques.

### Ontology design patterns

To provide definitions for the relations between classes that are used in the GENIA corpus, we developed two closely related ontology design patterns [32]. They are particularly suited for applications in text mining where the exact referent of a term cannot always be reliably determined. However, the patterns could be useful in other domains and applications as well.

The first ontology design pattern is applicable when a class *C* with the subclasses *D*_1_, ..., *D_n_* stands in a relation *CC-R* to a class *E* such that every instance of at least one subclass of *C* stands in a relation *II-R* to some instance of *E*. This pattern is useful when one class cannot be entirely disambiguated, and a superclass is used in a relation statement instead. For example, GGP classes in GENIA are primarily introduced because it is not always possible – or reasonable – to disambiguate entirely whether a term refers to *DNA*, *RNA* or *Protein* classes. Instead, the GGP class is used in relation statements, and the GGP class unifies the classes of *DNA*, *RNA* and *Protein*. In many cases, the relation is only relevant for the instances of one of the subclasses, e.g. only the *Proteins*, such that some property or relation applies to every instance of this subclass but not to all the instances of the other subclasses.

The specialized pattern for a relation *GGP-subclass-R* is as follows:(10)

The pattern in formula 10 can be further generalized, as it still uses the classes *DNA*, *RNA* and *Protein*. In terms of a class *C* with subclasses *D*_1_, ..., *D_n_* whose instances are standing in a relation *II-R* to some instance of *E*, the pattern is formulated as follows (where *R* is the relation between the two classes):(11)

The second ontology design pattern is derived from the definitions of the *has-variant* relations. It is applicable when every instance of a GGP class is related by the relation *II-S* either to some instance *x* of a class *D*, or to some individual which stands in a combination of the relations **T_1_**, ..., **T_m_** to *x*. The general pattern is as follows:(12)

In general, it is possible to consider either an order defined on the relations *T*_1_, ..., *T_m_* or arbitrary permutations. Intuitively, the pattern is used to state that all instances of one general class (the GGP class in the case of GGP annotations) stand in a relation *II-S* to some instance of a class *D* or to any entity reachable by a chain (or permutation) of the relations *T*_1_, ..., *T_m_* from any instance of this class.

### Future research

Although the formalization of relationships used in the GENIA annotation is itself valuable to provide a means for automated inference and verification as well as the development of annotation guidelines, formalized relations will be much more useful in combination with a formal characterization of *events*[8]. Events include more dynamic entities such as the *binding* of a molecule to a binding site. In conjunction with the formalization of the relations, more useful inferences would become possible. For example, from the assertion that a class *X binds Y* which is a *GGP-part-of Z*, we would be able to infer that *X GGP-binds Y*.

We propose ontology design patterns that are not limited to relations between GGPs but can be applied in many domains. For example, the patterns can be used to formally distinguish between functions and the processes that realize them when using the functional abnormality pattern [33,34]. We intend to explore further areas of application beyond the domain of genes and their products.

## Conclusions

We presented and discussed a formal ontology-based characterization of the relations used for annotating the GENIA corpus. The main challenge was the ambiguity of the terms upon which the relations are based. These terms refer to one of several ontological classes, and the definitions of the relationships between two terms had to reflect that only one of these classes can stand in some relation to another class. To characterize this phenomenon formally, we introduced the notion of a *GGP class*, which is an ontological class with subclasses whose names are not distinguishable within a certain annotation task. In our GENIA use case, the GGP class is a common superclass for classes of DNA, RNA and proteins, and is intended to unify classes of genes and their products.

We introduced two ontology design patterns to formally define relations that hold between a GGP class and another class. The ontology design patterns are especially useful whenever it is not possible – or not feasible – to determine the exact class that stands in some relation to another class, and a more general class is chosen in a relation statement instead. Therefore, they can be generalized to other domains and applications besides corpus annotation.

We implemented the axioms and definitions as well as the ontology design patterns in a software application that converts annotated GENIA abstracts into OWL ontologies. These ontologies can then be used to answer queries, verify annotations or provide a basis for knowledge-based machine learning techniques. Formalizing the relations used in the relationship annotations of the GENIA corpus provides a powerful means to verify the annotations, to reason over them and to establish and communicate unambiguous and precise annotation guidelines. The ontology of relations, its axioms and our ontology design patterns are applicable and useful beyond GENIA. They can be integrated in other ontology- or knowledge-based resources whenever two classes are considered to be indistinguishable and need to be disambiguated through automated reasoning.

## Authors' contributions

RH and AN conceived of the idea to formalize relations in GENIA and developed the formalism, SP and TO provided the intended meaning of relations and the description of the annotation process, SP, TO and AO contributed to formalization, RH implemented the software, DRS supervised the project, RH, AN, SP drafted the initial manuscript, all authors critically revised the manuscript.

## Competing interests

The authors declare that they have no competing interests.
